# Assessment of Availability of Antibiotics for Online Sale and Comparison of E-pharmacies in India

**DOI:** 10.7759/cureus.89577

**Published:** 2025-08-07

**Authors:** Puneet Kaur, Navjot Kaur, Jasmeen Kaur, Manvir Kaur, Jasbir Singh

**Affiliations:** 1 Pharmacology, Government Medical College, Patiala, IND; 2 Public Health, Johns Hopkins Bloomberg School of Public Health, Maryland, USA

**Keywords:** antibiotic awareness, online sale, overuse and misuse of antibiotics, pharmacies, who aware classification

## Abstract

Introduction

The global rise in antimicrobial resistance (AMR) poses a serious public health threat, reducing the effectiveness of commonly used antibiotics against prevalent bacterial infections. The mushrooming of Indian e-pharmacies, especially during and after the COVID-19 pandemic, has improved public access to medicines, including antibiotics. However, the ease of availability, combined with an ambiguous regulatory framework governing e-pharmacies, may inadvertently encourage the irrational use of antibiotics. Despite this growing concern, there is a paucity of data on the availability of antibiotics for online sale in India. Similarly, there is a lack of comparative analysis of e-pharmacies on key characteristics influencing safe and rational use of antibiotics.

Methods

The present study aims to assess the availability of antibiotics for online sale and compare the e-pharmacies on key characteristics influencing safe and rational use of antibiotics. A cross-sectional study was conducted over a six-month period, assessing 50 Indian e-pharmacies for the online availability of antibiotics, using the National List of Essential Medicines, 2022 and WHO-AWaRe (Access, Watch, Reserve) classification. Three customised questionnaires, namely, 'E-Pharmacy Characteristics', 'Adequacy of Antibiotic Product Information' and 'Consumer Awareness Information' questionnaire, were developed after a thorough review of available literature. These tools were used to collect information on parameters pertaining to a) safety and authenticity of e-pharmacies, b) availability of adequate drug information, and c) consumer awareness information available on websites for safe and responsible use of antibiotics. Ethical approval was not sought as the data were available in the public domain. The data analysis was conducted using descriptive statistics.

Results

A total of 17 antibiotics were studied. Antibiotics from all WHO-AWaRe categories were available for sale online, with a predominance of 'Watch' category antibiotics, followed by 'Reserve' and 'Access' categories. None of the e-pharmacies fully complied with the parameters outlined in the three questionnaires. Considerable variation was observed in their adherence to these parameters. For instance, while the majority of e-pharmacies had a customer support/grievance redressal policy (90%) and required a prescription for antibiotic sales (82%), none mentioned the registration status of the pharmacist responsible for answering queries or had measures in place to prevent excessive antibiotic orders.

Conclusion

Increased access to 'Watch' and 'Reserve' antibiotics may contribute to their overuse, which may potentially fuel growing AMR. The findings highlight the necessity for a stringent regulatory framework for e-pharmacies and emphasize the importance of consumer education on the safe and responsible use of antibiotics.

## Introduction

The World Health Organisation (WHO) has identified antimicrobial resistance (AMR) as one of the top global public health and development threats [1]. In 2019, bacterial AMR was directly responsible for 1.27 million deaths globally and 297,000 deaths in India [2]. The rising AMR situation in India is driven by several interconnected factors, including irrational use of antibiotics in human medicine and agriculture, inadequate infection prevention and control (IPC) measures, over the counter (OTC) sale of antibiotics without prescriptions in rural sectors and online due to weak regulatory enforcement, and lack of knowledge regarding its use among the general public [3,4]. In India, an ‘e-pharmacy’ has been defined as a business of distribution or sale, stock, exhibit, or offer for sale of drugs through a web portal or any other electronic mode [5]. Currently, there are around 50 e-pharmacies in India, and the e-pharmacy market in India is expected to rise from $0.5B (2019) to $4.5 B by 2025 [6]. While online platforms facilitate easier and cheaper access to medicines, including antibiotics, for the public, the lack of standardisation among different online pharmacies and the risk of counterfeit medications may compromise patient safety and antimicrobial stewardship (AMS) efforts [7,8]. Thus, the WHO highlights the need for regulation of online medicine distribution to ensure its safe and effective delivery to genuine patients [9]. The WHO has also developed the AWaRe (Access, Watch, and Reserve) classification of antibiotics to promote rational use of antibiotics and curb AMR [10]. Furthermore, one of the core objectives under the National Action Plan on Antimicrobial Resistance (NAP-AMR) in India is the enforcement of stringent regulatory measures to identify unlicensed pharmacies and prohibit the sale of antimicrobials as OTC drugs [11]. The Indian National List of Essential Medicines (NLEM) 2022 groups the drugs, including antibiotics, that should be available at different tiers of healthcare delivery to promote rational use [12]. The Indian regulatory system governing e-pharmacies is still evolving, as the draft e-pharmacy rules, 2018, have not been implemented yet, leading to continued legal ambiguity and operational challenges for e-pharmacies [13]. There is a paucity of data on both the online availability of antibiotics in India and the information provided by e-pharmacy platforms to support the prudent use of antibiotics.

To the best of our knowledge, there has been no similar analysis in the past. Therefore, the present study aims to analyse the availability of antibiotics through e-pharmacies in the context of the NLEM 2022 and WHO-AWaRe 2023 antibiotic categories and to compare e-pharmacies based on information provided on key characteristics that influence the safe and rational use of antibiotics.

This study was previously presented as a meeting abstract at the 2025 American Society for Pharmacology and Experimental Therapeutics (ASPET) Annual Meeting on April 3, 2025, and was awarded second place under 'Division of Translational and Clinical Pharmacology' in poster presentation.

## Materials and methods

This was a cross-sectional analysis conducted over a period of six months from April to October 2024. The popular e-pharmacies in India were searched using the keywords ‘buy antibiotics online in India’ on the Google search engine, and the top 50 results retrieved were selected [14]. Availability of a total of 17 antibiotics was assessed by conducting searches on the e-pharmacy websites. These were selected to represent antibiotics belonging to all “WHO-AWaRe” antibiotic categories that are available for use in India as per NLEM 2022. The list included six Access (amoxicillin, amoxicillin+clavulanic acid, cefazolin, clindamycin, cotrimoxazole, and doxycycline), six Watch (cefixime, ceftriaxone, piperacillin+tazobactam, meropenem, azithromycin, and vancomycin), and five Reserve (colistin IV, colistin oral, faropenem, linezolid, and tigecycline) antibiotics.

The selected websites were analysed to collect relevant information affecting the safe and rational use of antibiotics using three questionnaires that had been previously developed and validated by our study team [15]. The first questionnaire, titled *‘*E-Pharmacy Characteristics*’*, focused on assessing the safety and authenticity of e-pharmacies. It included questions pertaining to the registration status of e-pharmacy with the central drug authority, compliance with various applicable rules, the requirement of a prescription for placing an order, etc. The second, the *‘*Adequacy of Antibiotic Product Information*’*, evaluated different domains related to drug product details such as pharmaceutical information, pharmacological information like drug uses or contraindications, and references for information posted etc. The third, the *‘*Consumer Awareness Information*’ *questionnaire, examined the information provided by e-pharmacies to consumers to promote the safe use of antibiotics, such as to avoid self-medication or sharing of antibiotics and to complete the full course of treatment, etc. Online pharmacies were expected to fully comply with the parameters (numbers 1 to 9 and 14) listed in the *'*E-Pharmacy Characteristics' questionnaire, in accordance with the draft e-pharmacy rules 2018 [5]. Since the study is based on the data available in the public domain, ethical approval was not sought. Data were summarised and presented using descriptive statistics.

## Results

**Figure 1:**
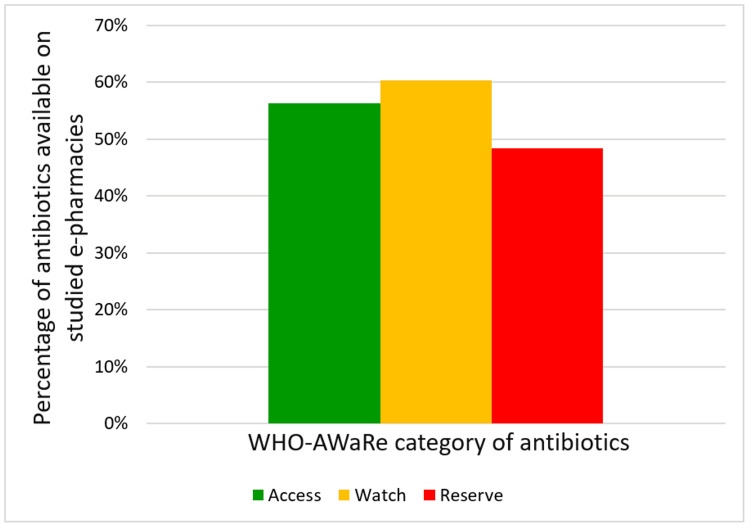
Availability of antibiotics on e-pharmacies Reference: [10]

Antibiotics, representative of all the WHO-AWaRe categories, were available for online sale, with a predominance of the Watch category (60.33%), followed by the Access (56.33%) and Reserve (48.40%) categories (Figure 1).

Although the majority of e-pharmacies provided a physical address/telephone number in country (96%), had a customer contact/grievance redressal policy (90%) and considered prescription as a must for placing an order (82%), but none of these e-pharmacies completely complied with the studied parameters, the details of which are summarized in Table 1.

**Table 1:** 'E-pharmacy Characteristics' questionnaire References: [5,15]

Q	Does the online/e-pharmacy portal provide	Websites (%)
1	Physical address and/or telephone number in the country?	96%
2	Any information regarding licensing with a state board of pharmacy or the Central Drug Standard Control Organization?	2%
3	Information regarding the constitution of the firm, including details of the directors, partners or persons having ownership of the e-pharmacy?	10%
4	Any quality certification logo?	8%
5	Return policy of dispensed drugs?	78%
6	Any reference to compliance with the following rules and subsequent amendments thereof:	
A	Drugs and Cosmetics Act, 1940	44%
B	Information Technology Act, 2000	60%
C	Consumer Protection Act, 1986	34%
D	Narcotic Drugs and Psychotropic Substances Act, 1985	4%
7	A licensed pharmacist on staff to answer consumer questions?	2%
8	Any information on the registration of the pharmacist?	None
9	Explicit information on the confidentiality of consumers’ personal or financial information?	82%
10	Require a prescription as a must for ordering antibiotics?	82%
11	Any information on a prescription is considered valid if it contains the date, patient’s details, the doctor’s details with sign?	20%
12	Any mechanism to prohibit the uploading of the same prescription on more than one e-pharmacy portal?	4%
13	Any information on keeping record of prescriber, patient and drugs dispensed in case of Schedule H1 drugs?	None
14	Customer support or grievance redressal facility?	90%

Furthermore, considerable differences were observed in compliance with key parameters included in the 'Adequacy of Antibiotic Product Information' questionnaire, such as the majority (76%) displaying basic information like brand name, dosage form, strength, etc., while very few of these provided scientific references (18%) or drug disposal information (4%). Other details of the study findings are presented in Table 2.

**Table 2:** 'Adequacy of Antibiotic Product Information' questionnaire Reference: [15]

Q1	Does the e-pharmacy portal provide information on:	Websites (%)
a)	Name of the active ingredient(s) using the approved generic name of the drug	72%
b)	Brand name	76%
c)	Dosage form	76%
d)	Dose strength	76%
e)	Name of excipients	42%
f)	Pack size information	74%
g)	Name of manufacturer, marketer and/or distributor	74%
h)	Expiry date	18%
i)	Drug storage conditions	34%
j)	Drug disposal	4%
Q2	Does the e-pharmacy portal mention	
a)	The drug as an antibiotic	64%
b)	How drug acts	56%
c)	How to take the drug	60%
d)	Approved therapeutic uses	62%
e)	Contraindications and warnings	50%
f)	Adverse drug reactions/side effects	58%
g)	Drug-drug interactions	26%
h)	Special population (e.g. Pregnancy, Lactation, Renal disease patients, Hepatic/Liver disease patients, elderly or children)	48%
Q3	Does the e-pharmacy portal	
a)	Provide the national pharmacovigilance number to report adverse drug reactions (if any)?	None
b)	Offer an alternative brand or class of antibiotics in case the requested antibiotic is unavailable?	40%
c)	Ask about any history of allergy, pregnancy or co-morbidities before placing the order?	None
Q4	Does the e-pharmacy portal provide	
a)	Any reference to scientific literature for the drug information provided?	18%
b)	Date on when was the drug information last updated?	12%
c)	Registration details of the author or reviewer of the drug information?	None

Notably, considerable discrepancies were observed pertaining to the 'Consumer Awareness Information' questionnaire, such as 60% of e-pharmacies advised to take medication as prescribed by the physician, while only a small proportion (2%) cautioned against self-medication or sharing antibiotics with others. Additional details are presented in Table 3.

**Table 3:** 'Consumer Awareness Information' questionnaire Reference: [15]

Q	Does the e-pharmacy portal	Websites (%)
1	Classify the antibiotic as per WHO-AWaRe classification?	None
2	Advise consumers to not to use antibiotics for viral infections?	14%
3	Advise consumers to take the medication exactly as prescribed by a physician?	60%
4	Advise consumers to avoid self-medication?	8%
5	Advise consumers to complete the full course of treatment?	44%
6	Advise consumers regarding consequences of not completing the antibiotic course?	44%
7	Advise consumers to consult physician in case of no relief or side effects?	50%
8	Advise consumers to avoid using leftover antibiotics?	2%
9	Advise consumers to avoid stockpiling of leftover antibiotics?	2%
10	Advise consumers to not to share their antibiotics with others?	2%
11	Advise consumers to not to take antibiotics prescribed for someone else?	None
12	Advise consumers to practise good hygiene for e.g. regular handwashing, proper food handling, and vaccination?	None

## Discussion

India has one of the highest rates of AMR globally [16]. Despite antibiotics being prescription-only medications, they are frequently available OTC at retail pharmacies, which can potentially exaggerate antibiotic misuse and overuse [16]. This nonprescription over-the-counter dispensing and potential easy purchase through online pharmacies enables patients to self-medicate without requiring a valid prescription. Such practices may also increase the risk of exposure to substandard and falsified medicines, especially antibiotics, which are among the most frequently counterfeited drug classes sold online [17]. To curb inappropriate use of antibiotics and improve access to the appropriate antibiotics, the WHO has developed the AWaRe classification tool.

Furthermore, the WHO recommends that at least 60% of total antibiotic use should be from Access group antibiotics [10]. The predominance of Watch category antibiotics (60.33%) as observed in our study is a cause of concern, especially so when our findings are substantiated by similar reports of availability of Watch category antibiotics on offline platforms as well [18]. These observations are not only contrary to WHO recommendations but also defeat the purpose of a tiered availability of antibiotics as per the NLEM to promote appropriate antibiotic use.

The rapid expansion of the pharmaceutical sector in India, including the mushrooming of e-pharmacies, has improved public access to antibiotics; however, the regulatory frameworks governing online pharmacies are still evolving [19]. As observed in our study, none of the e-pharmacies fully complied with the studied parameters across the three questionnaires. These included only a limited number of e-pharmacies mentioning regulatory registration status (2%), having a registered pharmacist on board (2%), displaying a quality certification logo (10%), etc. This reflects a general lack of adherence to requirements listed in the Indian draft e-pharmacy rules, 2018 [5]. In developed economies, such as the USA, there are programs such as Verified Internet Pharmacy Practice Sites (VIPPS), which serve as certification systems for the legitimation of e-pharmacies [20]. In Europe and Great Britain, certified e-pharmacies are required to display quality assurance logos signalling compliance with regulatory standards [21,22].

Despite antibiotics being prescription-only medicines, only 82% of e-pharmacies required a prescription prior to placing an order in the first place. Secondly, only 20% e-portals mentioned valid prescriptions. The ease of obtaining prescription medications without proper verification fosters self-medication and misuse, compromising patient safety and contributing to antibiotic resistance and related health complications [23]. The lack of scientific references to the information provided on the webpages of the majority of portals is another major concern. Given the increasing public reliance on the internet for health information, the provision of credible, referenced content on e-pharmacy platforms is critical to support informed healthcare decisions [24]. A recent systematic review with data from 77 studies across 24 low- and middle-income countries revealed that only 34.5% of the general public had adequate knowledge regarding AMR. This lack of awareness serves as one of the key drivers for AMR propagation and transmission. The WHO recognises the need for enhancing awareness and understanding of AMR, coupled with expert-led behavioural interventions to effectively combat this public health threat [25,26]. Similarly, the Centres for Disease Control and Prevention (CDC) has also initiated multiple public awareness programs on the responsible use of antibiotics [27].

We strongly advocate for the development of educational modules that should be made widely accessible to the public via e-pharmacies to foster the same in India. Furthermore, patient-reported data can offer valuable real-world insights into drug safety and contribute meaningfully to regulatory surveillance [28]. Since none of the e-pharmacies displayed information on reporting of adverse drug reactions (if any), we advocate for the inclusion of national pharmacovigilance contact details on e-portals for added patient safety. A limitation of our analysis is that it is based solely on information available online. In addition, since we did not procure any antibiotics from the e-pharmacies, we cannot comment on issues related to product quality, the ordering process, logistics services, etc.

## Conclusions

Easy public access to watch and reserve group of antibiotics through e-pharmacies can contribute to antibiotic overuse that may act as a catalyst for growing AMR. The study results call for urgent addressal of regulatory ambiguities by the Indian drug regulator. Stringent regulatory oversight coupled with consumer awareness programs regarding appropriate antibiotic use can go a long way in curbing the emerging AMR and safeguarding public health.
